# Amitriptyline Activates TrkA to Aid Neuronal Growth and Attenuate Anesthesia-Induced Neurodegeneration in Rat Dorsal Root Ganglion Neurons

**DOI:** 10.1097/MD.0000000000003559

**Published:** 2016-05-06

**Authors:** Xiaochun Zheng, Feng Chen, Ting Zheng, Fengyi Huang, Jianghu Chen, Wenshao Tu

**Affiliations:** From the Department of Anesthesiology (XZ, FC, TZ, FH, JC, WT), Provincial Clinical Medical College, Fujian Medical University, Fujian Provincial Hospital; and Fujian Provincial Emergency Center (FC), Provincial Clinical Medical College, Fujian Medical University, Fuzhou, China.

## Abstract

Tricyclic antidepressant amitriptyline (AM) has been shown to exert neurotrophic activity on neurons. We thus explored whether AM may aid the neuronal development and protect anesthesia-induced neuro-injury in young spinal cord dorsal root ganglion (DRG) neurons.

The DRG explants were prepared from 1-day-old rats. The effect of AM on aiding DRG neural development was examined by immunohistochemistry at dose-dependent manner. AM-induced changes in gene and protein expressions, and also phosphorylation states of tyrosine kinases receptor A (TrkA) and B (TrkB) in DRG, were examined by quantitative real-time polymerase chain reaction and western blot. The effect of AM on attenuating lidocaine-induced DRG neurodegeneration was examined by immunohistochemistry, and small interfering RNA (siRNA)-mediated TrkA/B down-regulation.

Amitriptyline stimulated DRG neuronal development in dose-dependent manner, but exerted toxic effect at concentrations higher than 10 M. AM activated TrkA in DRG through phosphorylation, whereas it had little effect on TrkB-signaling pathway. AM reduced lidocaine-induced DRG neurodegeneration by regenerating neurites and growth cones. Moreover, the neuroprotection of AM on lidocaine-injured neurodegeneration was blocked by siRNA-mediated TrkA down-regulation, but not by TrkB down-regulation.

Amitriptyline facilitated neuronal development and had protective effect on lidocaine-induced neurodegeneration, very likely through the activation of TrkA-signaling pathway in DRG.

## INTRODUCTION

Amitriptyline (AM), a tricyclic antidepressant, was recently identified to have potent neurotrophic activity through the activation of tyrosine kinases receptor A (TrkA) and B (TrkB).^1^ Most recently, AM was shown to exert neurotrophin-like effect to improve neuronal survival and cognitive functions in animal models of neurodegenerative diseases.^2–4^ In addition, clinical studies also demonstrated that AM treatment had neurotrophic effect on human brains.^5,6^ Thus, these collective studies all point to a potential therapeutic role of AM to act as a pro-neuronal reagent to stimulate neuronal growth, protect neurodegeneration, or promote neural regeneration in human neuro-diseases.

Dorsal root ganglion (DRG) is an important component in spinal cord to relay neural signaling from peripheral sensory systems to the brain, thus playing critical roles in neural development, neuropathic pain, and neurodegeneration in human diseases.^7–9^ Studies have shown that Trk receptors, including TrkA and TrkB, were actively regulated in DRG after spinal cord injury,^10,11^ and neurotrophic factors, such as nerve growth factor (NGF) or brain-derived neurotrophic factor (BDNF) played important role in DRG development, degeneration, and regeneration.^12–14^ However, it is not known whether AM may directly act on Trk receptors in DRG, or whether such actions may exert neurotrophic activities to facilitate neuronal development or promote neuro-regeneration in DRG.

Lidocaine is one of the commonly used anesthetics in clinics. Studies have demonstrated that lidocaine may induce neurodegeneration in spinal cord DRG.^15–17^ It was reported that neurotrophic factors might facilitate the recovery process of DRG neurons after lidocaine-induced injury.^18^ However, no study has shown direct evidence of AM facilitating neurotrophin-signaling pathway to counter lidocaine-induced neurotoxicity in DRG.

Therefore, first in this study, we cultured postnatal 1-day-old rat DRG in vitro and examined the effect of AM on aiding DRG neuronal development through the method of immunohistochemistry. We then used biochemical assays to analyze the expression of TrkA and TrkB in AM-treated DRG. In a neurodegeneration model, we explored whether AM might facilitate the recovery of DRG neurons after lidocaine-induced neurite loss and growth-cone collapse. Finally, we used small interfering RNA (siRNA) technology to specifically down-regulate TrkA or TrkB gene to investigate whether they were directly involved in the process of AM-mediated neural rescue after lidocaine injury.

### Ethics, Consent, and Permissions

In this study, all animal protocols were approved by the Clinical Research and Ethics Committee at Fujian Provincial Hospital, Provincial Clinical Medical College and Fujian Medical University in Fuzhou, Fujian Province in China.

## METHODS

### Cell Culture

In this study, DRGs were extracted from neonatal (postnatal 1 day) rats and cultured in vitro according to the methods described before.^19^ Briefly, DRGs were treated with Dulbecco Modified Eagle Medium (DMEM; ThermoFisher Scientific) and trypsin (0.5%; ThermoFisher Scientific) for 30 minutes at 37°C, followed by centrifugation at 850 rpm for 5 minutes. After discarding the supernatant, the ganglion pellet was resuspended in DMEM/F-12 medium (ThermoFisher Scientific) and triturated by 20 μL Eppendorf pipette for 5 minutes. The ganglion-containing medium was centrifuged again at 850 rpm for 5 minutes. The DRG pellet was then resuspended in 6-well plate containing DMEM/F-12, 10% fetal bovine serum (FBS; ThermoFisher Scientific), penicillin/streptomycin (PenStrep, ThermoFisher Scientific), and 20% neurobasal medium (ThermoFisher Scientific), and maintained in a cell-culture incubator with 95% O_2_ and 5% CO_2_ at 37°C.

### Immunohistochemistry

The culture medium was aspirated from 6-well plate. DRG culture was quickly fixed with 4% paraformaldehyde (ThermoFisher Scientific) in phosphate-buffer solution (PBS; ThermoFisher Scientific) for 10 minutes, and incubated with blocking solution containing 5% normal horse serum (ThermoFisher Scientific) and 0.1% Triton (Sigma-Aldrich) for 1 hour. DRG was then incubated with a mouse monoclonal Tuj-1 primary antibody (Santa Cruz) for 24 hours at 4°C, followed by a goat-antimouse Alexa Fluor 594 secondary antibody (ThermoFisher Scientific) for 2 hours at room temperature. The 6-well plate was then mounted on an inverted fluorescent microscopy system and examined under 10× objective with a TRTIC filter (Axio Observer A1, Zeiss, Germany). In each well, the lengths of tetraethyl rhodamine isothiocyanate-positive neurites were measured and averaged among 50 longest ones. The relative neurite growth was then quantified by normalizing the averaged neurite length for each experimental condition against the averaged neurite length under control condition.

### Quantitative Real-time Polymerase Chain Reaction

Total RNA was extracted from DRG culture using a Tri-reagent Kit (Molecular Research Center). The reverse transcription was conducted using an AccuPower RT-Premix kit (Bioneer, Republic of Korea). The quantitative real-time polymerase chain reaction (qRT-PCR) was conducted using a SYBR Green qRT-PCR kit (Applied Biosystems, CA) on an ABI Prism 7000 real-time PCR system (Applied Biosystems, CA) according to the manufacturer's protocol. 18S gene was the loading control. Relative mRNA amount for target gene was calculated as fold changes using 2^(^−^ΔΔCt)^ method.

### Western Blotting Analysis

Protein was extracted from DRG culture using a lysis buffer containing 1% Triton X-100, 50 mM HEPES, pH 7.5, 150 mM NaCl, 10% glycerol, 1.5 mM MgCl_2_, 1 mM EGTA, 100 mM sodium fluoride, 100 mM sodium phosphate, 10 μg/mL aprotinin, 10 μg/mL leupeptin, 1 mM phenylmethylsulfonyl fluoride, and 1 mM sodium pervanadate. For each sample, total protein of 35 μg was separated using 8% sodium dodecyl sulfate polyacrylamide gel electrophoresis and transferred to polyvinylidene fluoride membranes. Membranes were blocked with 5% nonfat dry milk in 1× Tris-buffered 0.05% Tween 20 solution (TBS-T; Sigma Aldrich) for 1 hour at room temperature. Membranes were then incubated with primary antibodies against TrkA (1:500; Santa Cruz), phospho-TrkA (1:250; Cell Signaling), TrkB (1:500; Santa Cruz), phospho-TrkB (1:250; Santa Cruz) overnight at 4°C. Membranes were washed with TBS-T (3× 10 minutes) and incubated with corresponding horseradish peroxidase-conjugated secondary antibodies (1:2000) for 2 hours at room temperature. After incubating with the Supersignal West Pico chemiluminescent kit (ThermoFisher Scientific) for 5 minutes, membranes were visualized using an enhanced chemiluminescence system (Pierce).

### Anesthesia Neurodegeneration and Rescue Assay

The assay of anesthetic reagent, lidocaine, induced neurodegeneration in DRG neurons, and also rescue by AM was conducted according to the method described previously.^18^ Briefly, DRG culture was treated with 5 mM lidocaine for 1 hour. Then, the culture medium was gently replaced with fresh medium without lidocaine, but with the addition of AM (0, 0.5, and 10 μM) for 24 hours. Immunohistochemical assay was then conducted and neurite growth was quantified.

### siRNA Assay

Chemically synthetized TrkA and TrkB-specific siRNAs, and a scrambled control siRNA (C-siRNA) were commercially obtained from RiboBio (RiboBio, Guangzhou, China). DRG culture was transfected with 100 nM siRNAs for 24 hours. The efficiency of specific gene down-regulation was each verified by qRT-PCR.

### Statistical Analysis

In this study, all assays were repeated for at least 3 times. Summarized data were presented as mean ± standard errors. Statistical significance was compared between summarized data using Student *t* test. *P* < 0.05 indicates significant difference.

## RESULTS

### Amitriptyline Aids Neuronal Development in DRG Neurons

**FIGURE 1:**
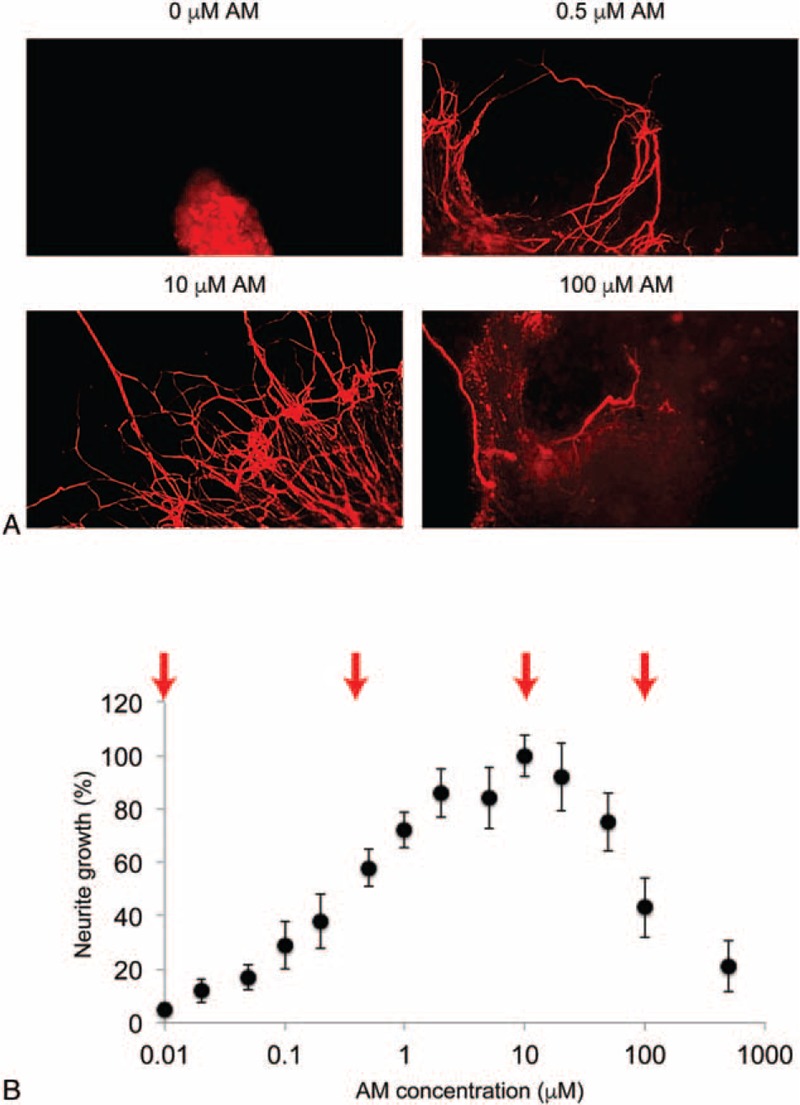
Amitriptyline (AM)-facilitated neuronal growth of DRG neurons. A, In postnatal 1-day-old rat, dorsal root ganglion (DRG) was cultured in 6-well plate and treated with different concentrations of AM (0, 0.5, 10, 100 μM) for 12 hours. Immunostaining of Tuj-1 antibody identified the neurites of DRG neurons in vitro. As compared with the treatment without AM (0 μM), there was moderate neurite outgrowth by 0.5 μM AM and significant neurite outgrowth by 10 μM AM. However, 100 μM induced toxicity in DRG neurons. B, AM-induced neuronal growth of DRG neurons was plotted in concentration-dependent manner. Four concentrations used in A were indicated (arrows). It showed AM aided neuronal growth of DRG neurons in concentration-dependent manner till 10 μM. After that, higher concentrations of AM had toxic effect on DRG neurons.

In the in vitro explant of neonatal (postnatal 1-day old) rat DRG, serial concentrations of AM were added for 12 hours. The immunohistochemistry assay of Tuj-1 staining was then conducted to identify the neurites of DRG neurons in the culture. It showed that, as compared with the condition without AM treatment (0 μM), 0.5 μM and 10 μM AM induced significant neurite growth in DRG neurons (Figure 1A). Interestingly, high concentration of AM (100 μM) had toxic effect on DRG neuronal growth (Figure 1A, panel of 100 μM AM). The quantification on DRG neurite growth showed that AM aided neuronal growth of DRG neurons in concentration-dependent manner (Figure 1B). However, the maximal effective concentration of AM to promote DRG neuronal growth was around 10 μM, as DRG neurons treated with higher concentrations of AM (>10 μM) had reduced neurite growth, likely due to the effect of high-dose drug toxicity (Figure 1B).

### Amitriptyline Activates TrkA but not TrkB in DRG Neurons

**FIGURE 2:**
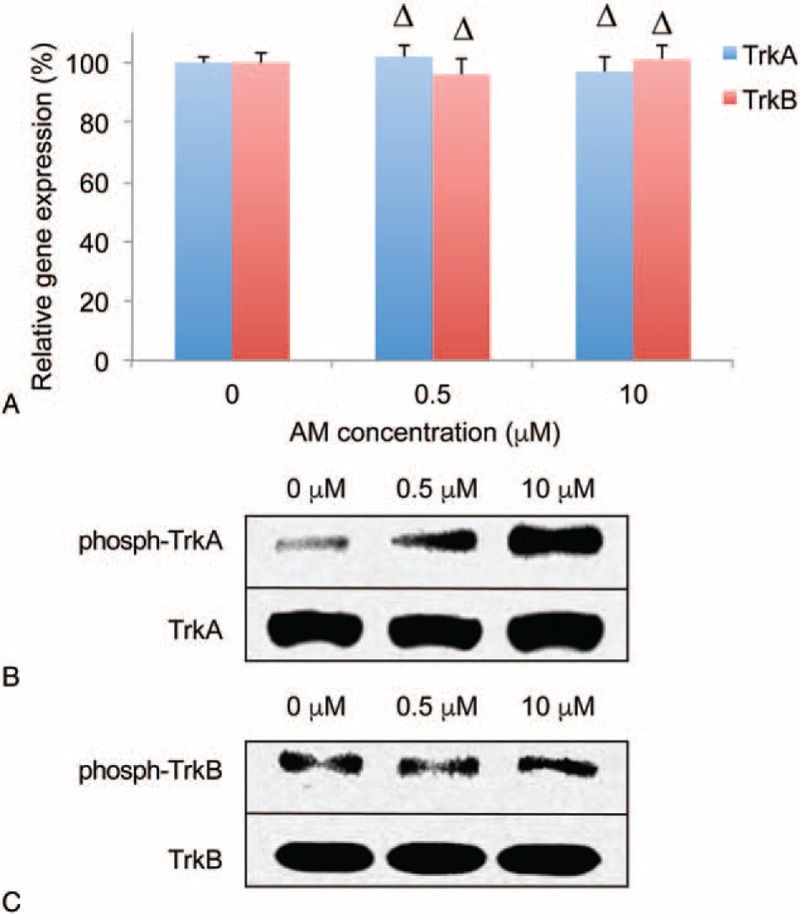
Amitriptyline (AM) activates TrkA in DRG neurons. A, DRG culture was treated without AM, or with AM (0.5 and 10 μM) for 12 hours. qRT-PCR was conducted to compare mRNA levels of TrkA and TrkB (Δ*P* > 0.05, vs 0 μM AM). B, Western blot was conducted to compare protein levels of TrkA, phosphorylated TrkA (phosph-TrkA), TrkB, and phosphorylated TrkA (phosph-TrkA). DRG = dorsal root ganglion, qRT-PCR = quantitative real-time polymerase chain reaction, TrkA = tyrosine kinases receptor A, TrkB = tyrosine kinases receptor B.

It was recently reported that AM acts as both TrkA and TrkB agonist.^1^ We thus wondered whether the facilitating effect of AM on neuronal growth of DRG neurons was mediated by the activation of either TrkA or TrkB (or both) signaling pathway(s). To examine this hypothesis, we conducted both qRT-PCR and western blot analysis to probe the effect of AM on TrkA and TrkB expression, and also their phosphorylation states in DRG culture. The results of qRT-PCR showed that, gene expression levels of TrkA and TrkB were little changed by AM in DRG neurons (Figure 2A; Δ*P* > 0.05, vs 0 μM AM), suggesting there were no endogenous autoup-regulation of TrkA or TrkB in DRG neurons. The results of western blot showed that while the protein levels of TrkA or TrkB were unchanged by AM, phosphorylated TrkA (phosph-TrkA) was significantly up-regulated by AM treatment (0.5 and 10 μM, vs 0 μM) (Figure 2B, top panel). Interestingly, phosphorylated TrkB (phosph-TrkB) was not up-regulated (or down-regulated) by AM treatment at either 0.5 or 10 μM (Figure 2B, bottom panel). These results indicate that AM activated TrkA through TrkA phosphorylation, whereas it had no effect on TrkB-signaling pathway, in DRG neurons.

### Amitriptyline Attenuates Lidocaine-induced DRG Neurodegeneration

Studies have demonstrated that, locally applied anesthetics, such as lidocaine, may cause growth-cone collapse and induce neurodegeneration in young DRG neurons.^16,18^ As we showed that AM activated TrkA-signaling pathways and aided neuronal development in DRG neurons, we wondered whether AM could protect lidocaine-induced neuro-injury in DRG neurons.

**FIGURE 3:**
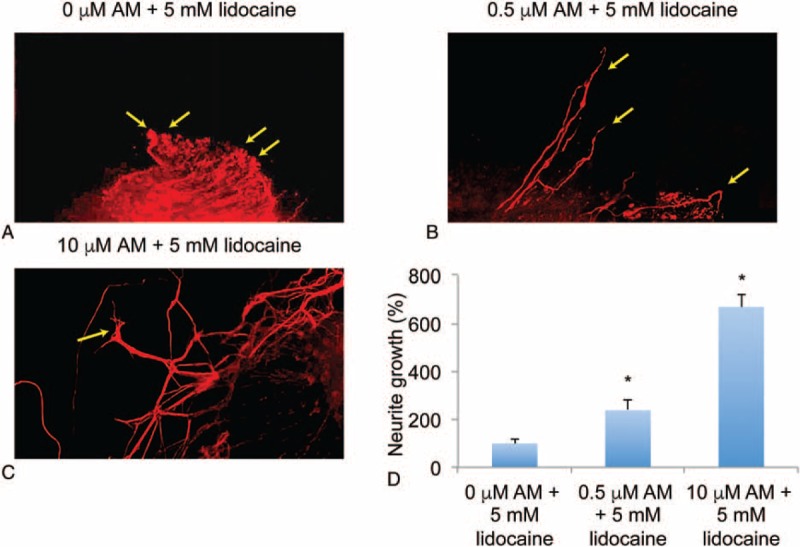
Amitriptyline (AM) regrew neurite and growth cone in lidocaine-injured DRG neurons. DRG culture was treated with 5 mM lidocaine for 1 hour. After medium replenishment, DRG was maintained for another 24 hours with the addition of AM (0, 0.5, or 10 μM). A, Immunohistochemical assay with Tuj-1 staining was conducted on DRG culture without AM treatment. Collapsed growth cones were identified (arrows). B, In lidocaine-injured DRG culture, then treated with 0.5 μM AM, regrown neurites were identified (arrows). C, In lidocaine-injured DRG culture, then treated with 10 μM AM, regrown growth cones were identified (arrows). D, AM-induced neuronal growth in DRG neurons after lidocaine-induced injury was quantified (^∗^*P* < 0.05, vs 0 μM AM). DRG = dorsal root ganglion.

We used a previously described model to induce growth-cone collapse and neurodegeneration in DRG neurons by in vitro application of lidocaine.^18^ DRG culture was treated with 5 mM lidocaine for 1 hour, followed by 24 hour culture with fresh medium with AM (0, 0.5, and 10 μM). The results of Tuj-1 immunostaining showed that lidocaine induced significant loss of DRG neurites and growth-cone collapses in DRG neurons (Figure 3A, arrows). However, while DRG neurons were treated with 0.5 μM AM for 24 hours, regrowth of neurites was observed (Figure 3B, arrows). Then, while DRG neurons were treated with 10 μM AM for 24 hours, not only neurite regrew, but also growth cones were newly regenerated in the culture (Figure 3C, arrows). Quantification on relative neurite growth confirmed our observation, showing that AM, at both 0.5 and 10 μM, stimulated significant neurite outgrowth (Figure 3D, ^∗^*P* < 0.05, vs 0 μM AM). Therefore, our immunohistochemical analysis indicates that AM had protective effect on lidocaine-induced neurodegeneration in DRG neurons.

### TrkA is Involved in Amitriptyline-mediated Protection on Lidocaine-induced DRG Neurodegeneration

**FIGURE 4:**
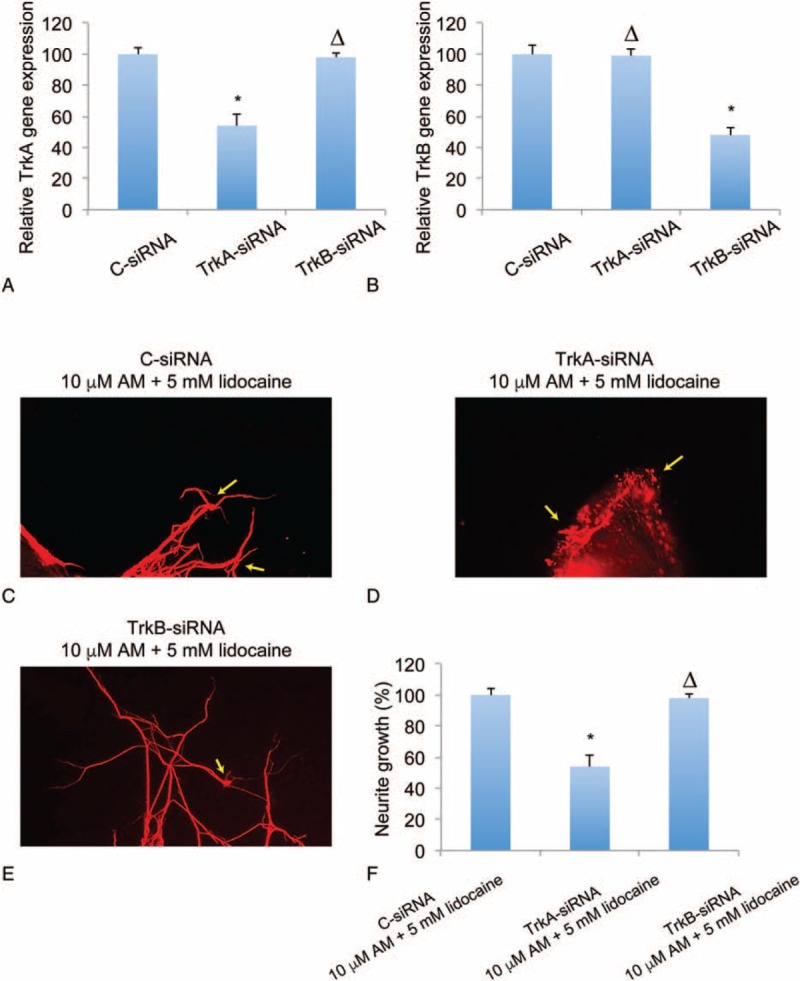
Down-regulation of TrkA blocked amitriptyline (AM)-mediated regrowth of lidocaine-injured DRG neurons. DRG culture was transfected with 100 nM TrkA-specific siRNA (TrkA-siRNA), TrkB-specific siRNA (TrkB-siRNA), or a control siRNA (C-siRNA) for 24 hours. qRT-PCR was conducted to compare the posttransfection mRNA expressions of TrkA (A) and TrkB (B) (^∗^*P* < 0.05, Δ*P* > 0.05). In DRG culture transfected with C-siRNA (C), TrkA-siRNA (D), or TrkB-siRNA (E), 5 mM lidocaine was added for 1 hour, followed by 24 hours incubation of 10 μM AM. Immunohistochemical assay with Tuj-1 staining was then conducted and growth cones were identified (arrows). F, The relative neurite growth in DRG neurons pretreated with siRNAs were compared (^∗^*P* < 0.05, Δ*P* > 0.05). DRG = dorsal root ganglion, qRT-PCR = quantitative real-time polymerase chain reaction, TrkA = tyrosine kinases receptor A, TrkB = tyrosine kinases receptor B.

Finally, we examined whether the attenuation of AM on lidocaine-induced DRG neurodegeneration was due to the activation of Trk receptors. We transfected with DRG culture with TrkA-siRNA or TrkB-siRNA, and also a C-siRNA (100 nM each) for 24 hours. Analysis of qRT-PCR showed that both TrkA-siRNA and TrkB-siRNA effectively and selectively knocked down its targeted genes (Figure 4A and B; ^∗^*P* < 0.05, Δ*P* > 0.05, vs C-siRNA). After 24 hours of siRNA transfection, we conducted the neurodegeneration assay followed by 24 hours treatment of 10 μM AM. Then, the results of Tuj-1 immunostaining showed that, in DRG culture pretransfected with C-siRNA or TrkB-siRNA, there was moderate regrowth of neurites and growth cones (Figure 4C and E, arrows). However, in DRG culture pretransfected with TrkA-siRNA, the neuronal regrowth was reduced and the collapsed growth cones, instead of regenerated ones, were found (Figure 4D, arrows). Quantification on relative neurite growth confirmed our observation, showing that TrkA-siRNA, and not TrkB-siRNA, reduced the AM-mediated outgrowth of DRG neurons after lidocaine-induced neurodegeneration (Figure 4F; ^∗^*P* < 0.05, Δ*P* > 0.05, vs C-siRNA). Therefore, our results strongly suggest that the protective effect of AM on lidocaine-induced neurodegeneration was through TrkA, not TrkB, signaling pathway in DRG neurons.

## DISCUSSION

Little is known about the direct effect of AM on the molecular mechanisms of spinal cord DRG. In a previous study, AM was shown to affect the electrophysiological properties of DRG neurons by blocking both tetrodotoxin-sensitive and tetrodotoxin-resistant Na^+^ currents,^20^ which may be closely associated with its traditional therapeutic effect of tricyclic antidepressant. In this study, we used a neonatal rat DRG explant to demonstrate that AM stimulated the neuronal development of DRG neurons by promoting neurite outgrowth. Thus, unlike the mechanism of AM blocking Na^+^ channels in previous finding, the underlying mechanisms of our findings are very likely involving distinctly different signaling pathways, probably those of newly discovered neurotrophin-like activities of AM.^1,4^ Interestingly, we also found that at high concentrations (>10 μM), AM induced neurite loss and possibly apoptosis among DRG neurons. This finding is in line with study in adult rat DRG showing apoptotic effect by high concentrations of AM, likely through mitochondrial dysfunction and activation of caspase proteins.^21^

We then explored AM-associated signaling pathways in DRG. We found that the gene and protein expression levels of TrkA and TrkB were unchanged by AM. These results suggested that AM did not exert pro-neuronal effects on DRG neurons by simply altering the endogenous expression of Trk receptors. Rather, as we discovered, AM activated TrkA-signaling pathway by phosphorylating TrkA protein. It was known that NGF, and also its receptor TrkA, are crucial for the development of DRG.^12,22^ Thus, it is very likely that AM-induced TrkA phosphorylation is the major contributor to stimulate neuronal growth in DRG neurons. Interestingly, we also found that TrkB-signaling pathway was not affected by AM, because levels of TrkB phosphorylation were similar among DRG neurons treated with or without AM. It is worth noting that the majority of neonatal DRG neurons expressed TrkA, not TrkB or TrkC.^23^ Thus, it is possible that, in our experiment, TrkA phosphorylation, instead of TrkB phosphorylation, was the major signaling pathway associated with AM due to predominant expression of TrkA.

We also demonstrated that AM facilitated DRG neuronal regeneration after lidocaine-induced neurotoxicity through the activation of TrkA-signaling pathway. In a recent study, Li et al^24^ reported that caspase proteins, including caspase-2 and caspase-3, were actively involved in lidocaine-induced DRG apoptosis. It is not clear whether TrkA activation is directly interacting with caspase-signaling pathways in lidocaine-injured DRG. However, study had shown that NGF activated TrkA-signaling pathway and regulated cJun N-terminal kinase 2 to reduce caspase-2/3 in olfactory bulb.^25^ Further study in DRG would certainly help to elucidate the possible association between TrkA and caspase-signaling pathways in regulating DRG neuro-injury.

## CONCLUSIONS

In conclusion, we presented clear evidence showing novel mechanisms of AM in stimulating neuronal growth and facilitating postinjury neuronal regeneration in DRG. We also demonstrated that AMs were very likely acting as neurotrophic agonist to activate TrkA, but not TrkB, signaling pathways in DRG. Thus, beyond its traditional role of an antidepressant, AM may also be considered as a novel therapeutic reagent for spinal cord injury or neurodegenerative disease.
